# Differential identification of *Mannheimia haemolytica* genotypes 1 and 2 using colorimetric loop-mediated isothermal amplification

**DOI:** 10.1186/s13104-023-06272-8

**Published:** 2023-01-19

**Authors:** Rohana P. Dassanayake, Michael L. Clawson, Fred M. Tatum, Robert E. Briggs, Bryan S. Kaplan, Eduardo Casas

**Affiliations:** 1 United States Department of Agriculture, National Animal Disease Center, Ruminant Diseases and Immunology Research Unit, Agricultural Research Service, Ames, IA 50010 USA; 2 United States Department of Agriculture, Agricultural Research Service, U.S. Meat Animal Research Center, Animal Health Genomic Research Unit, Clay Center, NE 68933 USA

**Keywords:** Adhesins, Genotypes, Loop-mediated isothermal amplification, LAMP, Leukotoxin, *Mannheimia haemolytica*, Serotypes

## Abstract

**Objective:**

*Mannheimia haemolytica* is the primary bacterial pathogen associated with bovine respiratory disease complex (BRDC). While *M. haemolytica* has been subdivided into 12 capsular serotypes (ST), ST1, ST2 and ST6 are commonly isolated from cattle. More recently, *M. haemolytica* strains isolated from North American cattle have been classified into genotypes 1 (ST2) and 2 (ST1 and ST6). Of the two genotypes, genotype 1 strains are frequently isolated from healthy animals whereas, genotype 2 strains are predominantly isolated from BRDC animals. However, isolation of both genotypes from pneumonic lung samples can complicate diagnosis. Therefore, the aim of this study was to develop a colorimetric loop-mediated isothermal amplification (LAMP) assay to differentiate *M. haemolytica* genotypes.

**Results:**

The genotype specificity of the LAMP was tested using purified genomic DNA from 22 M*. haemolytica* strains (10 genotype 1, 12 genotype 2) and strains from four related *Pasteurellaceae* species; *Bibersteinia trehalosi*, *Mannheimia glucosida*, *Pasteurella multocida*, and *Histophilus somni*. Genotype 1 (adhesin pseudogene B1) specific-LAMP reactions amplified DNA only from genotype 1 strains while genotype 2 (adhesin G) reactions amplified DNA only from genotype 2 strains. The overall detection sensitivity and specificity of the newly developed colorimetric LAMP assay for each genotype were 100%. The limits of detection of two LAMP assays were 1–100 target gene copies per reaction. LAMP primers designed in this study may help the differential identification of *M. haemolytica* genotypes 1 and 2.

**Supplementary Information:**

The online version contains supplementary material available at 10.1186/s13104-023-06272-8.

## Introduction

*Mannheimia haemolytica* (formerly *Pasteurella haemolytica*) is a Gram negative, opportunistic pathogen that primarily resides as a commensal in the nasopharynx and tonsil regions of the upper respiratory tracts of healthy cattle and other ruminants [1]. *M. haemolytica* is the primary bacterial pathogen associated with bovine respiratory disease complex (BRDC), commonly known as shipping fever, causing extensive economic losses to the beef and dairy cattle industries in the United States and worldwide [2–4]. When the host’s immune system is compromised, *M. haemolytic*a can descend to the lower respiratory tract with a concomitant development of pneumonia [5]. BRDC is an acute respiratory infection characterized by the development of necrotizing, fibrinous-, broncho- or pleuro-pneumonia often associated with death, particularly in recently weaned beef calves shortly after entry to feedlots [5].

*M. haemolytica* can be classified into 12 capsular serotypes (ST) based on rapid plate agglutination or indirect hemagglutination tests using anti-capsular sera [6–8]. *M. haemolytica* ST2 is commonly isolated from healthy animals while ST1 and ST6 are largely isolated from lungs of animals affected with BRDC [1, 5]. However, isolation of all three serotypes from samples collected from animals suffering from BRDC can occur and complicate diagnosis [1]. Furthermore, Angen et al., (1999) suggested that serotyping does not always represent a reliable method to identify *M. haemolytica* or *Mannheimia glucosida* isolates [6]. Recently, *M. haemolytica* isolated from North American cattle were classified into two genotypes, 1 and 2, based on multiple SNP allele differences and the presence or absence of several genes, such as outer membrane proteins, a peptidase S6, a ligand-gated channel, an autotransporter outer membrane beta-barrel domain-containing protein, a porin, and three different trimeric autotransporter adhesins that were specific to genotype 2 as their homologs were either pseudogenes or completely absent in genotype 1 [9, 10]. *M. haemolytica* genotype 1 is primarily represented by ST2 strains while genotype 2 is primarily represented by ST1 and ST6 strains [9, 10].

Notomi et al., (2000) developed a novel nucleic acid amplification method, LAMP, that amplifies DNA rapidly with high specificity under isothermal conditions [11]. The LAMP reaction uses a specific DNA polymerase with a strand displacement activity along with four primers which recognize six specific target sequences on template DNA and amplifies them at isothermal conditions [11]. The LAMP reaction can be accelerated by the addition of two loop primers [12]. Since 2000, the LAMP method has emerged as an important and versatile diagnostic technique to detect various pathogens [13]. Multiple detection methods have been used to determine LAMP amplicons such as agarose gel with DNA-binding dyes, turbidity detection with turbidity meter, real-time fluorescence detection with fluorescent dyes, and colorimetric detection with phenol red or hydroxynaphthol blue [11, 12, 14].

Although capsular serotyping is still the gold standard to identify *M. haemolytica* serotypes, serotyping is not always accurately identify *M. haemolytica* or *M. glucosida* isolates [6]. Furthermore, the lack of access to capsular-specific sera for many laboratories can further limit serotyping capabilities [6]. In addition to serotyping, molecular tools such as conventional PCR, real-time PCR and pulsed-field gel electrophoresis are available to identify *M. haemolytica* strains, as are a MALDI-TOF assay and culture phenotyping characterizations [15–18]. Recently, a LAMP assay was developed to identify BRDC pathogens, however, the primer sets described for *M. haemolytica* appear to be only species-specific and is unable to discriminate serotypes or genotypes [19]. Therefore, the goal of this study was to develop a LAMP-based assay to distinguish *M. haemolytica* genotype 1 from genotype 2 strains.

## Materials and methods

### Bacterial strains, serotyping, and genomic DNA purification

A total of 22 M*. haemolytica* strains (genotype 1 = 10 and genotype 2 = 12; samples were collected from BRDC animals from the states of Kansas, Kentucky, Missouri, and Tennessee in 2013) and four related *Pasteurellaceae* species *Bibersteinia trehalosi* (n = 1), *Mannheimia glucosida* (n = 1), *Pasteurella multocida* (n = 1), and *Histophilus somni* (n = 1) were used in this study. Most of the *M. haemolytica* strains used in this study were molecularly serotyped by PCR in a previous study [9] and the remaining non-serotyped strains used here were serotyped by rapid plate agglutination test with serotype-specific rabbit antisera generated against ST1 and ST6 as described previously [8]. Bacterial isolates were grown in trypticase soy agar supplemented with 5% sheep blood (Becton, and Dickinson Co., Sparks, MD) at 37 °C in a humidified atmosphere of 7.5% CO_2_ for 16 to 48 h. Genomic DNA of each strain was purified using DNeasy Blood & Tissue Kit as per manufacturer’s instructions (Qiagen Inc, Valencia, CA). DNA concentration was determined by measuring the absorbance at 260 nm wavelength using a spectrophotometer.

### Design of genotype specific LAMP primers

*M. haemolytica* genotype 1 adhesin pseudogene B1 (GenBank accession no. CP017495.1, locus tag BG548_01640), genotype 2 adhesin G (GenBank accession no. CP017538.1, locus tag BG586_06285), and leukotoxin D (*lktD*; GenBank accession no. CP005972.1, locus tag F382_07400) nucleotide sequences were used to design LAMP primers. All the primers were designed using NEB online LAMP Primer Design Tool (https://lamp.neb.com). Details of primers are shown in Additional file 2: Table S1. They also were checked against the adhesin pseudogene B1 sequences of 36 genotype 1 M*. haemolytica* strains and the adhesin G sequences of 45 genotype 2 M*. haemolytica* strains, respectively, with complete genomes in GenBank for variation within primer sites with none detected (Additional file 2). *M. haemolytica rsmL* specific LAMP primer set used in this study was described elsewhere [19]. All the primers were synthesized by Integrated DNA Technologies (IDT Inc., Coralville, IA).

### Colorimetric LAMP assay

Colorimetric LAMP reactions were performed using a pH-based WarmStart® colorimetric LAMP 2 × master mix (with UDG) and a non-pH-based WarmStart® multi-purpose LAMP/RT-LAMP 2 × master mix (with UDG) containing *Bst* 2.0 DNA Polymerase (NEB Inc., Ipswich, MA). LAMP reactions were carried out using clear PCR 8-tubes strips in a 25 µl final volume containing 12.5 µl 2 × master mix, 2.5 µl 10 × primer mix (2 µm F3 and B3, 16 µm FIP and BIP, and 4 µm LF and LB, Additional file 2: Table S1), 1 µl purified genomic DNA (5 ng) and 9 µl nuclease-free water. A final concentration of 120 µm hydroxynaphthol blue (CAS No. 63451–34-4; Santa Cruz Biotechnology Inc., Dallas, TX) was added to the non-pH-based LAMP master mix as described previously [14]. Tubes were placed in a thermocycler and incubated at 65 °C for 60 min. A real-time fluorescence detection was also performed with a multi-purpose LAMP kit (LAMP fluorescent dye provided with the kit) using QuantStudio 5 Real-Time PCR system in MicroAmp Optical 96-well reaction plates (ThermoFisher Scientific, Waltham, MA). Briefly, LAMP reactions were carried out using Optical 96-well plates in a 25 µl final volume containing 12.5 µl of WarmStart® multi-purpose LAMP/RT-LAMP 2 × master mix, 2.5 µl of 10 × LAMP primer mix, 0.5 µl of 50 × fluorescent dye (provided with the kit), 1 µl of genomic DNA, and 8.5 µl nuclease-free water. Plates were incubated at 65 °C for 60 min and fluorescence readings (SYBR Green I filter) were recorded every 30 s. To further confirm the colorimetric results, LAMP products were electrophoresed on 1% (w/v) agarose gels and stained with SYBR-Safe DNA gel stain.

### Sensitivity of LAMP assay

Sensitivity analysis of LAMP primers was performed using multi-purpose LAMP master mix supplemented with hydroxynaphthol blue. Briefly, LAMP reactions were carried out using clear PCR 8-tubes strips in a 25 µl final volume containing 12.5 µl of WarmStart® multi-purpose LAMP/RT-LAMP 2 × master mix, 2.5 µl of 10 × LAMP primer mix, 0.5 µl of 6 mM hydroxynaphthol blue (Santa Cruz Biotechnology Inc., Dallas, TX; CAS 63451–35-4; final concentration = 120 µM), 1 µl of genomic DNA, and 8.5 µl nuclease-free water. The genomic DNAs from one genotype 1 strain and two genotype 2 strains were used here. DNA concentration in nanograms per microliter was converted to copies per microliter using ThermoFisher Scientific ‘DNA Copy Number and Dilution Calculator’. Genomic DNA was 10 × serially diluted in nuclease-free water and (10^0^–10^5^ copies per microliter) used for LAMP reaction. PCR 8-tubes strips were placed in a thermocycler and incubated at 65 °C for 60 min.

## Results and discussion

**Fig. 1:**
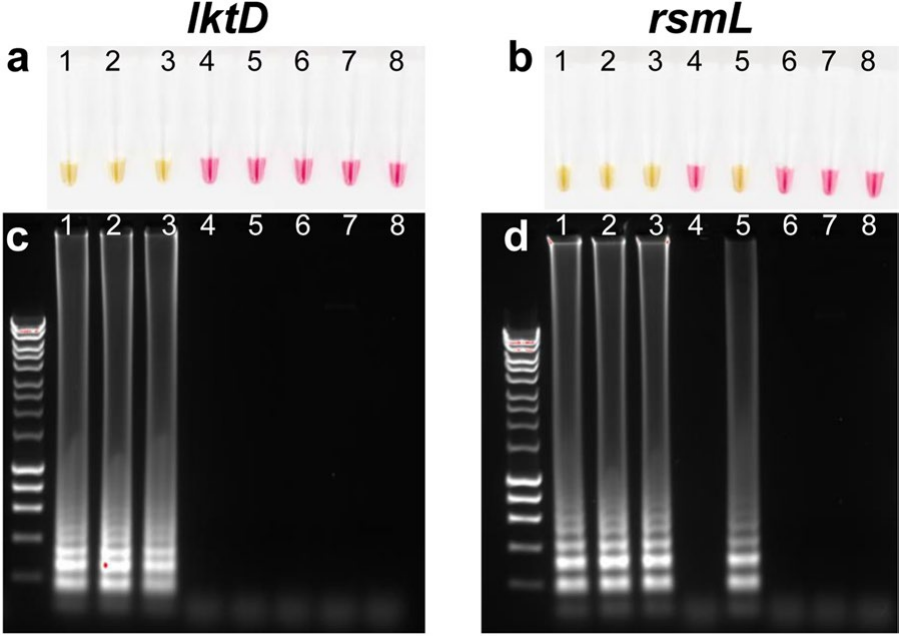
Comparison of *Mannheimia haemolytica* species-specificity of *lktD* and *rsmL* LAMP primers. Colorimetric LAMP reactions were performed with *lktD* (**a**) and *rsmL* (**b**) primer sets using a pH-based colorimetric LAMP kit (phenol red) with the purified genomic DNA (5 ng) from *M. haemolytica* genotypes 1 and 2 strains and four related *Pasteurellaceae* species at 65 °C for 60 min. A positive reaction is indicated by a color change from pink to yellow. LAMP amplicons obtained with *lktD* (**c**) and *rsmL* (**d**) primers were further analyzed by agarose gel electrophoresis. Tubes/lanes information: 1. *M. haemolytica* genotype 2 (ST1, D153); 2. genotype 1 (ST2, D171); 3. genotype 2 (ST6, D174); 4. *Bibersteinia trehalosi*; 5. *Mannheimia glucosida*, 6. *Pasteurella multocida*; 7. *Histophilus somni*; 8. No template control. In each gel, molecular weight marker (Bioline HyperLadder 1 kb) is shown to the left of lane 1

A variety of nucleic acid amplification methods, such as polymerase chain reaction (PCR), nucleic acid sequence-based amplification (NASBA), self-sustained sequence replication (3SR), and rolling circle amplification (RCA) are available. However, LAMP assay is gaining popularity as a point-of-care and other diagnostic applications due to its simplicity and amplification of DNA/RNA with high specificity within 15–60 min under isothermal conditions without the need for thermocycler [11]. Furthermore, positive reaction can be visually determined without agarose gel electrophoresis when using a pH-based and a non-pH-based colorimetric indicators such as phenol red and hydroxynaphthol blue in the reaction mixture, respectively [11, 14]. Since LAMP is a simple, rapid, and sensitive assay, our first goal was to design *M. haemolytica* species-specific LAMP primers targeting the well-conserved *lktD* gene of the leukotoxin operon [20]. Purified genomic DNAs from *M. haemolytica* genotype 2 (strains D153 and D174) and genotype 1 (strain D171) were initially compared with four related *Pasteurellaceae* species; *B. trehalosi*, *M. glucosida*, *P. multocida*, and *H. somni*, since they also are opportunistic pathogens that reside as commensals of the upper respiratory tract of cattle [21, 22]. The colorimetric LAMP with *lktD* primers showed a positive detection (indicated by a color change from pink to yellow) only with *M. haemolytica* strains and not with the related *Pasteurellaceae* species (Fig. 1a, lanes 4–7). Although *rsmL* LAMP produced positive results for *M. haemolytica*, it also showed a positive result for *M. glucosida* (Fig. 1b). The observation of ladder-like banding patterns on agarose gels with the positive LAMP samples including *M. glucosida* (which was amplified with *rsmL* but not *lktD* primers) confirmed the colorimetric LAMP results (Fig. 1c, d). These findings suggested that *lktD* (but not *rsmL* LAMP primers) can be used to identify *M. haemolytica*.

**Fig. 2:**
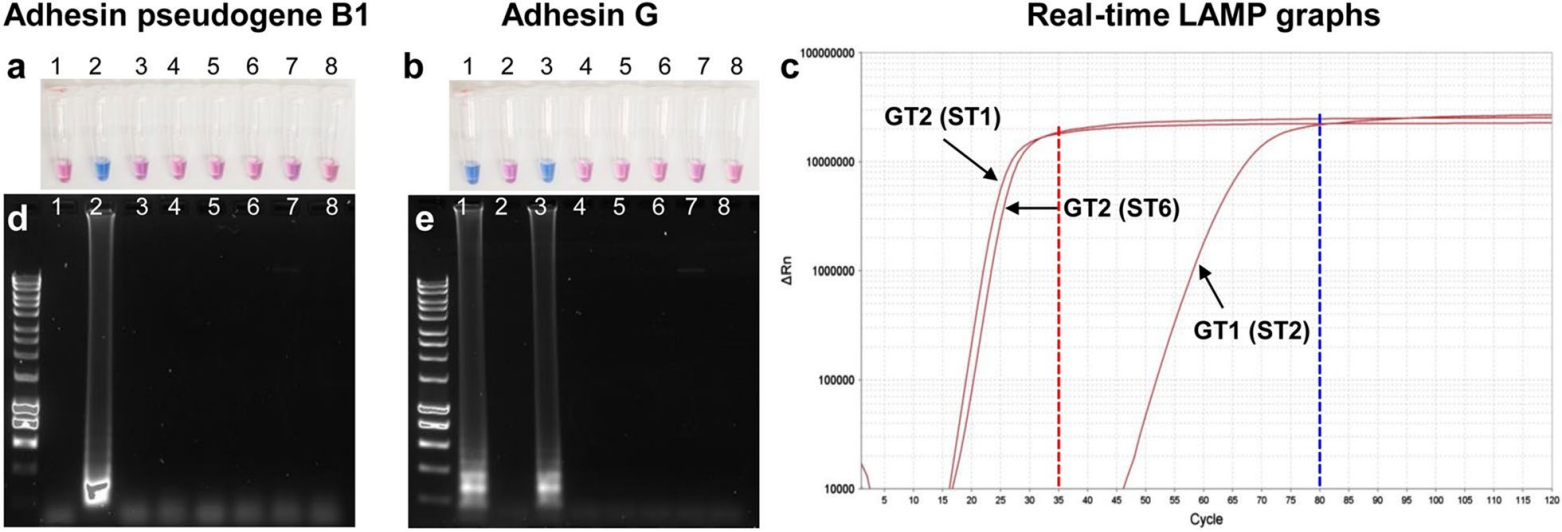
Colorimetric and real-time LAMP detection of *Mannheimia haemolytica* genotypes with genotype-specific primers. Colorimetric LAMP reactions were performed using adhesin pseudogene B1 (**a**, genotype 1) and adhesin G (**b**, genotype 2) specific primer sets using a non-pH-based multi-purpose LAMP kit supplemented with hydroxynaphthol blue with the purified genomic DNA (5 ng) from *M. haemolytica* genotypes 1 and 2 strains and four related *Pasteurellaceae* species at 65 °C for 60 min. A positive reaction is indicated by a color change from violet to sky blue. Similarly, a real-time LAMP amplification of genotype 1 strain (ST2: D171) and genotype 2 strains (ST1: D153 and ST6: D174) was performed with LAMP fluorescent dye at 65 °C for 60 min. Fluorescence readings (SYBR Green I) were recorded every 30 s (**c**). LAMP amplicons obtained with adhesin pseudogene B1 (**a**) and adhesin G (**b**) primers were further analyzed by agarose gel electrophoresis, (**d**) and (**e**), respectively. Tubes/lanes information: 1. *M. haemolytica* genotype 2 (ST1, D153); 2. genotype 1 (ST2, D171); 3. genotype 2 (ST6, D174); 4. *Bibersteinia trehalosi*; 5. *Mannheimia glucosida*, 6. *Pasteurella multocida*; 7. *Histophilus somni*; 8. No template control. In each gel, molecular weight marker (Bioline HyperLadder 1 kb) is shown to the left of lane 1. GT1: genotype 1, GT2: genotype 2

Although multiple LAMP assays are available to detect pathogens relevant to food animals, only one study has been conducted so far to detect BRDC bacterial pathogens [19]. In that study, the *M. haemolytica* LAMP was species-specific and was unable to discriminate serotypes and genotypes [19]. Furthermore, under our experimental conditions, *rsmL* LAMP primers also amplified *M. glucosida* genomic DNA suggesting a lack of specificity of *rsmL* primers for *M. haemolytica.* The second goal of this study was to develop LAMP primers to discriminate genotypes 1 and 2. We used the adhesin pseudogene B1 and the adhesin G gene, which have been observed in genotype 1 and genotype 2 strains, respectively [9], to design LAMP primers for genotype discrimination. A positive reaction with hydroxynaphthol blue in the colorimetric LAMP assay was indicated by a color change from violet to sky blue. As expected, adhesin pseudogene B1 primers produced positive LAMP results only with a genotype 1 strain (Fig. 2a) while adhesin G primers produced positive LAMP results only with two genotype 2 strains (Fig. 2b). No amplification (as indicated by no color change) was observed with related *Pasteurellaceae* species (Fig. 2a, b). The observed ladder-like patterns on agarose gels only in lanes with positive LAMP samples further confirmed the colorimetric LAMP findings (Fig. 2d, e). The lack of adhesin gene amplification with *H. somni* and *P. multocida* genomic DNA was expected since neither adhesin pseudogene B1 nor adhesin G were detected in the genomes of both species in a recently completed study [23].

To determine how early amplification was completed, a real-time LAMP assay was performed using a multi-purpose LAMP kit supplemented with a LAMP fluorescent dye. Analysis of real-time data revealed that genotype 2 specific LAMP primers showed increased fluorescence signal after ~ 17 cycles (~ 8.5 min) and maximum signal after ~ 35 cycles (17.5 min; red dotted-line, Fig. 2c). However, increased signal for genotype 1 specific LAMP primers was at ~ 47 cycles (~ 23.5 min) and maximum signal was at ~ 80 cycles (~ 40 min; blue dotted-line, Fig. 2c). Although genotype 2 LAMP primer set has both loop forward and loop backward primers, only one loop primer for genotype 1 could be generated. Therefore, the apparent delay in amplification of genotype 1 might be attributed to the lack of one loop primer. Similar observations have been previously reported for loop primers [12].

To further confirm the genotype-specificity of primers, we performed LAMP assay with nine genotype 1 and ten genotype 2 M*. haemolytica* strains which were previously characterized for adhesin genes [9]. Based on the previous study, all nine genotype 1 strains used in this study were ST2 [9]. Five of the previously untyped genotype 2 strains were serotyped by rapid plate agglutination test in this study for ST1 and ST6. Two of the five strains were identified as ST1 while remaining three were identified as ST6. Representative colorimetric LAMP results of eight strains for each genotype are shown in shown in Additional file 1: Fig. S1. As predicted, genotype 1 primers were specific to genotype 1 strains while genotype 2 primers were specific to genotype 2 (Additional file 1: Fig. S1).

**Fig. 3:**
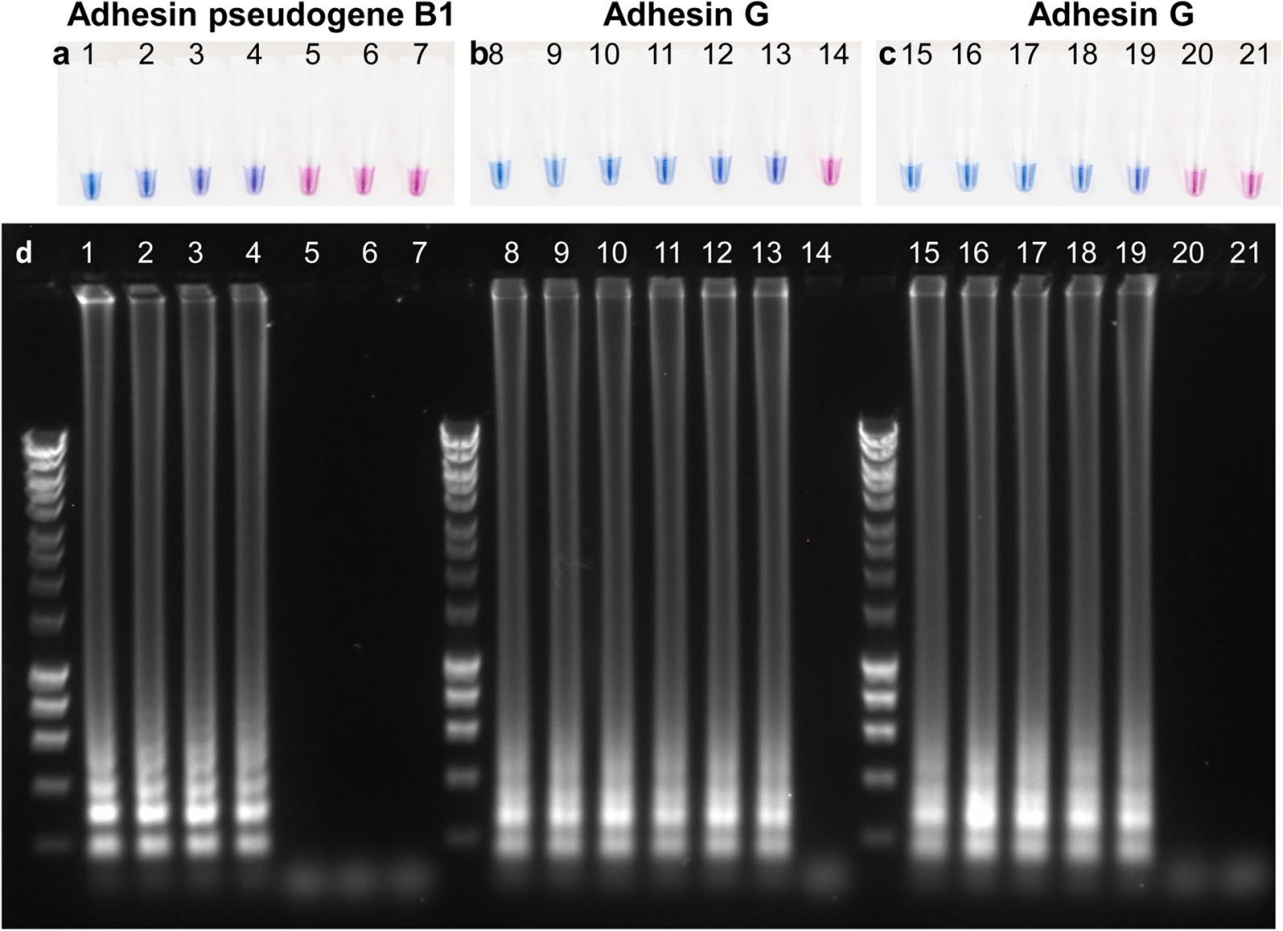
Colorimetric LAMP to determine the sensitivity of *Mannheimia haemolytica* genotype-specific primers. Sensitivity of LAMP primer sets designed for *M. haemolytica* genotype 1 (**a**, adhesin pseudogene B1, strain 22604 [ST2]) and genotype 2 (**b**, **c**; adhesin G, strains 32635 [ST1] and 33041 [ST6]) were performed using a non-pH-based multi-purpose LAMP kit supplemented with hydroxynaphthol blue with 10 × serially diluted genomic DNA at 65 °C for 60 min. A positive reaction is indicated by a color change from violet to sky blue. LAMP amplicons obtained with adhesin pseudogene B1 and adhesin G were also analyzed by agarose gel electrophoresis (**d**). Tubes/lanes information: 1. genotype 1 [ST2] 10^5^ copies; 2. 10^4^ copies; 3. 10^3^ copies; 4. 10^2^ copies; 5. 10^1^ copies; 6. 10^0^ copies; 7. No template control; 8. genotype 2 [ST1] 10^5^ copies; 9. 10^4^ copies; 10. 10^3^ copies; 11. 10^2^ copies; 12. 10^1^ copies; 13. 10^0^ copies; 14. No template control; 15. genotype 2 [ST6] 10^5^ copies; 16. 10^4^ copies; 17. 10^3^ copies; 18. 10^2^ copies; 19. 10^1^ copies; 20. 10^0^ copies; 21. No template control. In each gel, molecular weight marker (Bioline HyperLadder 1 kb) is shown at left to the lane 1, 8 or 15, respectively

Next, we examined the sensitivity of genotype-specific primers by colorimetric LAMP with the purified genomic DNA from one genotype 1 and two genotype 2 strains. Genotype 1 specific primers were able to detect 100 copies per reaction (Fig. 3a) while genotype 2 specific primers were able to detect 1 copy to 10 copies per reaction (Fig. 3b, c). Agarose gel electrophoresis of LAMP products were consistent with colorimetric LAMP findings (Fig. 3d).

Taken together, these results indicate that colorimetric LAMP assay can be used to rapidly discriminate *M. haemolytica* genotype 1 and 2. Therefore, genotype-specific LAMP should assist in proper diagnosis of BRDC.

## Limitations

Only purified genomic DNA from 22 M*. haemolytica* genotypes 1 and 2 strains were included in LAMP reaction tests. The LAMP primers were sensitive enough to detect 1–100 copies of targets for three strains used in this study, however, examination of additional samples may reveal differences in sensitivity. Additionally, only three of the twelve *M. haemolytica* serotypes and four related *Pasteurellaceae* species were tested for *lktD* and genotype LAMP primers for specificities for *M. haemolytica*. The LAMP assay is useful for genotyping single isolates of *M. haemolytica* but should be tested more before we can recommend its use on samples of mixed DNA from multiple organisms.

## Supplementary Information

**Additional file 1: Fig. S1.** Colorimetric LAMP results of *Mannheimia haemolytica* with genotype-specific primers. Colorimetric LAMP reactions were performed using adhesin pseudogene B1 (**a**, **b**; genotype 1) and adhesin G (**c**, **d**; genotype 2) specific primer sets using a pH-based (**a**,** c**; phenol red) and non-pH-based (**b**, **d**; hydroxynaphthol blue) LAMP kit with the purified genomic DNA (5 ng) from *M. haemolytica* genotypes 1 and 2 (8 strains, each) at 65°C for 60 minutes. A positive reaction is indicated by a color change from pink to yellow (**a**, **c**) or violet to sky blue (**b**, **d**). GT1: genotype 1; GT2: genotype 2; NTC: no template control.

**Additional file 2: Table S1.** Primers used in *Mannheimia haemolytica* species-specific and genotype-specific LAMP reactions.

**Additional file 3: Table S2.** Mannheimia haemolytica strains used to check for variation in LAMP primer hybridization sites.

## Data Availability

The datasets used and/or analyzed during the current study are in the manuscript and any additional details will be made available from the corresponding author on reasonable request.

## Abbreviations

BRDC: Bovine respiratory disease complex
GT: Genotypes
LAMP: Loop-mediated isothermal amplification
FIP: Forward inner primer
BIP: Backward inner primer
F3: Forward outer primer
B3: Backward outer primer
LF: Loop forward primer
LB: Loop backward primer
*M. haemolytica*: *Mannheimia haemolytica*
ST: Serotypes
