# Supplementary material for: Differential identification of Mannheimia haemolytica genotypes 1 and 2 using colorimetric loop-mediated isothermal amplification
Source: BMC Res Notes. 2023 Jan 19;16:4. doi: 10.1186/s13104-023-06272-8 (PMC9850709; doi:10.1186/s13104-023-06272-8)
Supplement: Supplementary file 1 — Additional file 1: Fig. S1. Colorimetric LAMP results of Mannheimia haemolytica with genotype-specific primers. Colorimetric LAMP reactions were performed using adhesin pseudogene B1 (a, b; genotype 1) and adhesin G (c, d; genotype 2) specific primer sets using a pH-based (a, c; phenol red) and non-pH-based (b, d; hydroxynaphthol blue) LAMP kit with the purified genomic DNA (5 ng) from M. haemolytica genotypes 1 and 2 (8 strains, each) at 65°C for 60 minutes. A positive reaction is indicated by a color change from pink to yellow (a, c) or violet to sky blue (b, d). GT1: genotype 1; GT2: genotype 2; NTC: no template control. [file 13104_2023_6272_MOESM1_ESM.docx]

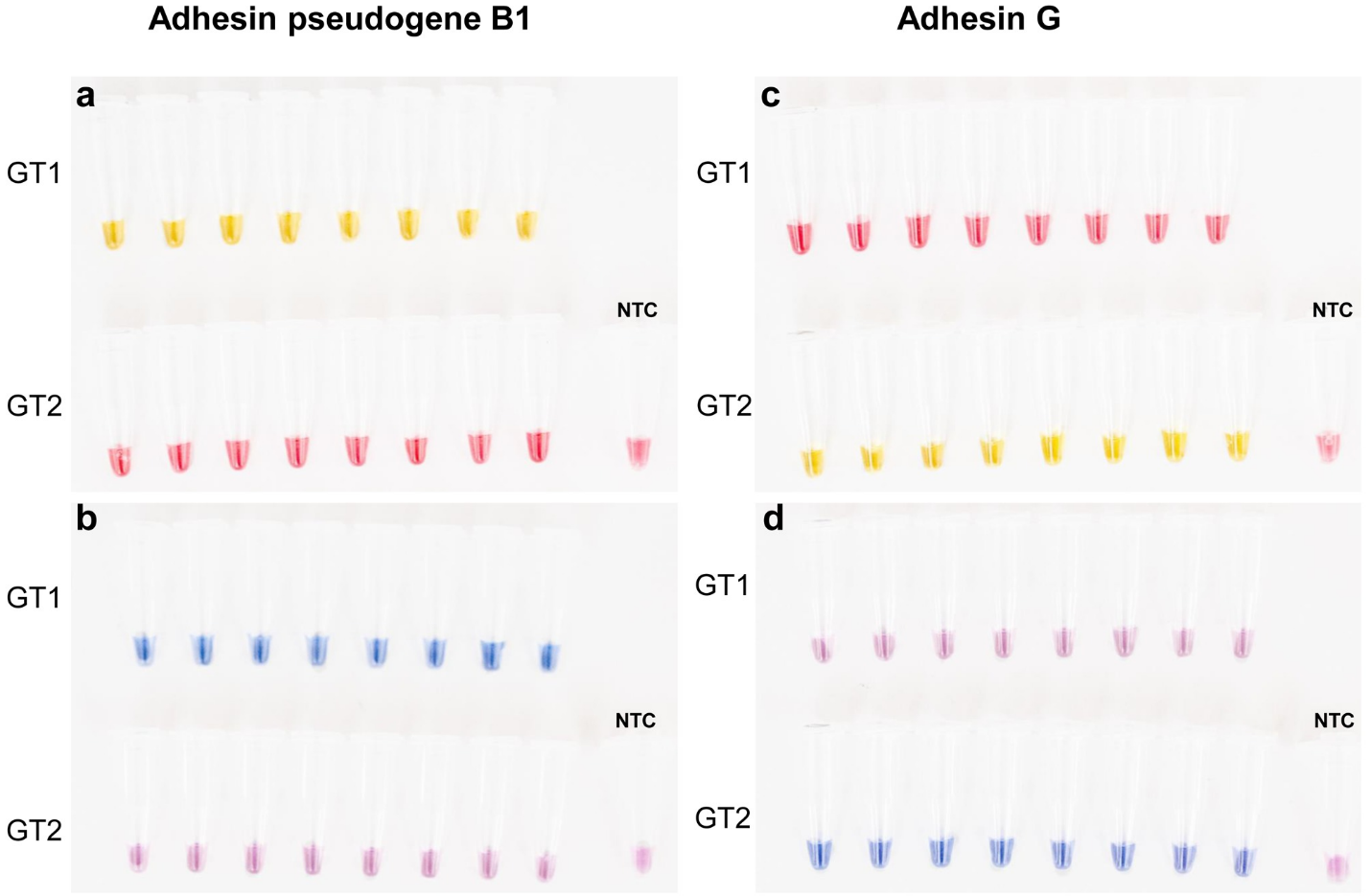


**Fig. S1** Colorimetric LAMP results of *Mannheimia haemolytica* with genotype-specific primers. Colorimetric LAMP reactions were performed using adhesin pseudogene B1 (**a**, **b**; genotype 1) and adhesin G (**c**, **d**; genotype 2) specific primer sets using a pH-based (**a**, **c**; phenol red) and non-pH-based (**b**, **d**; hydroxynaphthol blue) LAMP kit with the purified genomic DNA (5 ng) from *M. haemolytica* genotypes 1 and 2 (8 strains, each) at 65°C for 60 minutes. A positive reaction is indicated by a color change from pink to yellow (**a**, **c**) or violet to sky blue (**b**, **d**). GT1: genotype 1; GT2: genotype 2; NTC: no template control.
