# Supplementary material for: Differential identification of Mannheimia haemolytica genotypes 1 and 2 using colorimetric loop-mediated isothermal amplification
Source: BMC Res Notes. 2023 Jan 19;16:4. doi: 10.1186/s13104-023-06272-8 (PMC9850709; doi:10.1186/s13104-023-06272-8)
Supplement: Supplementary file 2 — Additional file 2: Table S1. Primers used in Mannheimia haemolytica species-specific and genotype-specific LAMP reactions. [file 13104_2023_6272_MOESM2_ESM.docx]

**Table S1.** Primers used in *Mannheimia haemolytica* species-specific and genotype-specific LAMP reactions.

**Primer set**

1. leukotoxin D Sequence (5` - 3` direction)

lktD-FIP GGTGGCCTCTAACACATCGTGTTACGACTGCTGAAACCT

lktD-BIP TGGTTCCAAACAAAGATATCGGCTCATAGCGTGTATAAGGGAAAG lktD-F3 ACTGAAAATTCACACTATAGGTG

lktD-B3 GCTAATATGTTTAATTCGACCAGTT

lktD-LF TCCGGCACAATGATCATCA

lktD-LB TTGCAGCAGGGCAGGAG

1. adhesin G

AdG-FIP ACCCGGTGCAACAAACTTAATTTGGTTGTTGATGGTGATTATGTGAG

AdG-BIP GCTTCCAATTCAACAGATGCGAGCCAGTTTTAACTAAAGTGTCAG

AdG-F3 GCACATTAAAATATAGCAGCTTTG

AdG-B 3 CTAAGCCAGAGTGATCCG

AdG-LF GCATTGGTTGCATTACCCA

AdG-LB GGTTCACAACTTTACGCAGT

3. adhesin pseudogene B1

pAdB1-FIP GAGCACCTGCTTTCGCTACACTGAAACAGGTGATAATGGTC

pAdB1-BIP AGAAGGCGATGAAAACAAAGTTATGACAGATGAGGTTTTCTCTGA

pAdB1-F3 CAGGTTTAGCAACTGTTGG

pAdB1-B3 AGTTACGGCTTTAGAACCA

pAdB1-LB ACCGCAGGTGATGTGGC
